# Monocytes Expressing IL‐36G Play a Crucial Role in Atopic Dermatitis

**DOI:** 10.1111/jcmm.70503

**Published:** 2025-03-30

**Authors:** Yitao Yang, Lei Wang, Longmei Yu, Chenxi Chang, Honglei Zhang, Linhan Hu, Juntong Liu, Yihang Zhang, Hui Han, Haiyun Zhang, Yumei Zhou, Ji Wang

**Affiliations:** ^1^ School of Medicine Shanghai University Shanghai China; ^2^ Hubei Shizhen Laboratory Hubei University of Chinese Medicine Wuhan China; ^3^ National Institute of TCM Constitution and Preventive Medicine Beijing University of Chinese Medicine Beijing China

**Keywords:** atopic dermatitis, cytokines, IL36G, immune infiltration, monocytes

## Abstract

Atopic dermatitis (ad) is a chronic inflammatory skin disease, with recent studies indicating that immune cells, such as monocytes and inflammatory cytokines, play a crucial role. By retrieving datasets from public databases and analysing immune cell infiltration in lesional skin using CIBERSORT, we found that monocytes and M2 macrophages were significantly upregulated in atopic dermatitis. Differentially expressed gene (DEG) functional enrichment analysis revealed that cytokine‐cytokine receptor interaction was the most significantly enriched pathway. Further analysis of cytokines and their receptors, along with their correlation with infiltrating immune cells, identified IL36G‐expressing monocytes as a key target in atopic dermatitis. We compared immune cell infiltration and cytokine‐related targets in similar inflammatory skin diseases, such as psoriasis and urticaria, to evaluate similarities and differences among these three skin conditions. The analysis revealed that IL36G‐expressing monocytes were also highly expressed in psoriasis but did not play a pivotal role in urticaria. Finally, we used molecular docking to predict and validate drugs targeting IL36G. Our study highlights IL36G‐expressing monocytes as a common key target in atopic dermatitis and psoriasis, offering novel insights and therapeutic strategies for these related diseases.

## 1 Introduction

Atopic Dermatitis (ad) is a common chronic inflammatory skin disease characterised by dry skin, itching and recurrent rashes, which significantly impact patients' quality of life [1, 2, 3]. The prevalence of ad is approximately 10% in adults, while it is as high as 15%–20% in children [4]. The pathogenesis of ad is complex, involving multiple factors such as genetic predisposition, immune system abnormalities, and environmental influences [5]. The characteristics of the ad phenotype include IgE‐mediated sensitisation, skin barrier dysfunction, and immune dysregulation leading to skin hypersensitivity to environmental triggers.

IL‐36 is a cytokine that plays a significant role in immune responses and inflammation, belonging to the IL‐1 family [6]. Three subtypes—IL‐36α, IL‐36β, and IL‐36γ—are primarily expressed in various cell types, including monocytes and macrophages. Among them, IL‐36G (Interleukin‐36 gamma) is particularly important in skin inflammation and immune responses, especially in autoimmune and inflammatory diseases [7]. IL‐36G is highly expressed in several inflammatory diseases and correlates with disease severity. Under conditions of infection, injury, or autoimmune diseases, monocytes produce increased levels of IL‐36G. Studies have shown that the mRNA expression of IL‐36G is significantly upregulated in the eczematous skin areas of patients with atopic dermatitis [8]. Research has shown that IL‐36G is highly expressed in the lesional skin of patients with acute generalised pustular psoriasis. In vitro, pathogenic drugs can specifically induce peripheral blood monocytes to directly release IL‐36G or, in the presence of autologous peripheral blood monocytes, induce its release from keratinocytes [9]. In patients with inflammatory bowel disease, IL‐36G is significantly increased in the infiltrated mucosa and is produced by T cells, monocytes/macrophages, and plasma cells [10]. IL‐36G exerts a pro‐inflammatory effect on primary human keratinocytes [11]. As key components of the inflammatory response, monocytes are involved in antigen presentation and the regulation of immune responses [12]. Monocytes play a crucial role in skin inflammation, and the inflammatory lesions in the skin depend on the expansion of activated monocytes and macrophages, as well as lymphatic drainage to the lymph nodes [13]. Additionally, monocytes are attracted to inflamed skin by various chemokines and cytokines [14]. Increased infiltration of monocytes is a hallmark of both acute and chronic inflammatory diseases [15]. When monocytes infiltrate the skin, they release various inflammatory mediators, such as cytokines and chemokines, which initiate and sustain local inflammation, resulting in symptoms like itching, redness, swelling, and skin damage. Monocytes interact with other immune cells, including T cells, B cells, and mast cells, to amplify or regulate immune responses, contributing significantly to the chronic inflammatory state in atopic dermatitis [16].

Atopic Dermatitis (ad), psoriasis (PSO) and chronic urticaria (CU) are clinically distinct inflammatory skin conditions. Psoriasis is a chronic, relapsing inflammatory disease, while urticaria is an acute skin reaction triggered by allergens. Despite the differences between ad and PSO, they share some common features, such as immune cell infiltration in the skin, altered expression of similar pro‐inflammatory cytokines, and changes in the skin barrier [17]. Although various treatment options are available, including topical medications, systemic therapies, and biologics, the effectiveness of atopic dermatitis treatments remains challenging due to individual differences and the diversity of the disease [18, 19]. Therefore, gaining a deeper understanding of its pathogenesis and related factors is of great significance for advancing research and improving clinical diagnosis and treatment.

This study, based on transcriptomic data from lesional skin, aims to identify key immune cells and therapeutic targets involved in atopic dermatitis lesions. Furthermore, it investigates the roles of these targets in similar inflammatory skin diseases, such as psoriasis and urticaria. The findings provide critical insights for the clinical diagnosis and treatment of atopic dermatitis.

## 2 Material and Methods

### 2.1 Transcriptome Data Source

We downloaded the atopic dermatitis RNA‐Seq dataset GSE224783, including 33 skin samples from 11 patients' acute, chronic and non‐lesional skin biopsies, from the GEO database detected by GPL16791. The raw gene expression data in psoriasis and controls were gathered from datasets GSE67785, which included 28 skin samples from psoriasis patients' lesions and uninvolved skin. For CU, we included 9 lesional skin samples and 12 non‐lesional skin samples from GSE72540. Finally, we downloaded the ad and psoriasis dataset GSE224783, including 10 ad lesional skins and 9 psoriasis lesional skins. Differentially expressed genes (DEGs) were screened using the ‘limma’ software package [20], and *p* < 0.05 and |logFC| > 1 were considered to indicate significant differences.

### 2.2 Functional Enrichment Analysis of DEGs

GO (Gene Ontology) and KEGG (Kyoto Encyclopedia of Genes and Genomes) enrichment analyses of DEGs were performed using the ‘clusterProfiler’ and ‘ggplot2’ package [21]. GSEA was performed on the gene expression matrix through the ‘clusterProfiler’ package, and the ‘c2.cp.kegg_legacy.v2023.2.Hs.symbols.gmt’ was selected as the reference gene set. Enrichment was statistically significant when *p* < 0.05.

### 2.3 Immune Infiltration Analysis

To assess the degree of immune cell infiltration in the skin, the CIBERSORT algorithm [22] was used to calculate the proportions of different immune cell types based on the expression levels of immune cell‐related genes. The ‘ggplot2’ package was further used to show the differences in 22 types of infiltrating immune cells between groups. Additionally, the Spearman method was employed to analyse the correlation between core biomarkers and the expression levels of infiltrating immune cells. The *p*‐values have also been adjusted using the same Benjamini–Hochberg method. A *p* < 0.05 was considered statistically significant.

### 2.4 Identification of Small Molecular Therapeutic Agents

The Broad Institute's Connectivity Map (cMAP) database (https://clue.io/) was used for the identification of small candidate molecules related to ad [23]. For the identification of small candidate chemical molecules, DEGs (|logFC| > 1) were introduced into the cMAP database for GSEA. The PubChem (https://pubchem.ncbi.nml.gov) was used for the extraction of detailed information and 3D confirmation of the established small molecules. Then, molecular docking experiments were carried out, and the crystal structures of key targets were retrieved from the human database in the PDB (https://www.rcsb.org/) database. AutoDockTool software was used to dewater and hydrogenate the receptor protein, and PyMOL 0.99rc6 software was used to perform molecular docking between small drug molecules and target proteins. A negative value indicates free binding. In this study, a binding energy value less than −5 kcal/mol was set as significant binding.

### 2.5 Statistical Analysis

R software (version 4.3.3) was used for statistical analysis. Data was shown as mean ± standard deviation (SD). The differences between the two groups were evaluated by student's *t*‐test with a result of *p* < 0.05 for statistical significance.

## 3 Results

### 3.1 IL‐36G‐Expressing Monocytes May Be a Hub Target in Atopic Dermatitis

To study DEGs in atopic dermatitis lesions, we downloaded the gene expression matrix from GSE224783 datasets. We screened for DEGs using the thresholds of *p* < 0.05 and |logFC| > 1, identifying 997 DEGs, including 509 upregulated and 488 downregulated genes.

**FIGURE 1:**
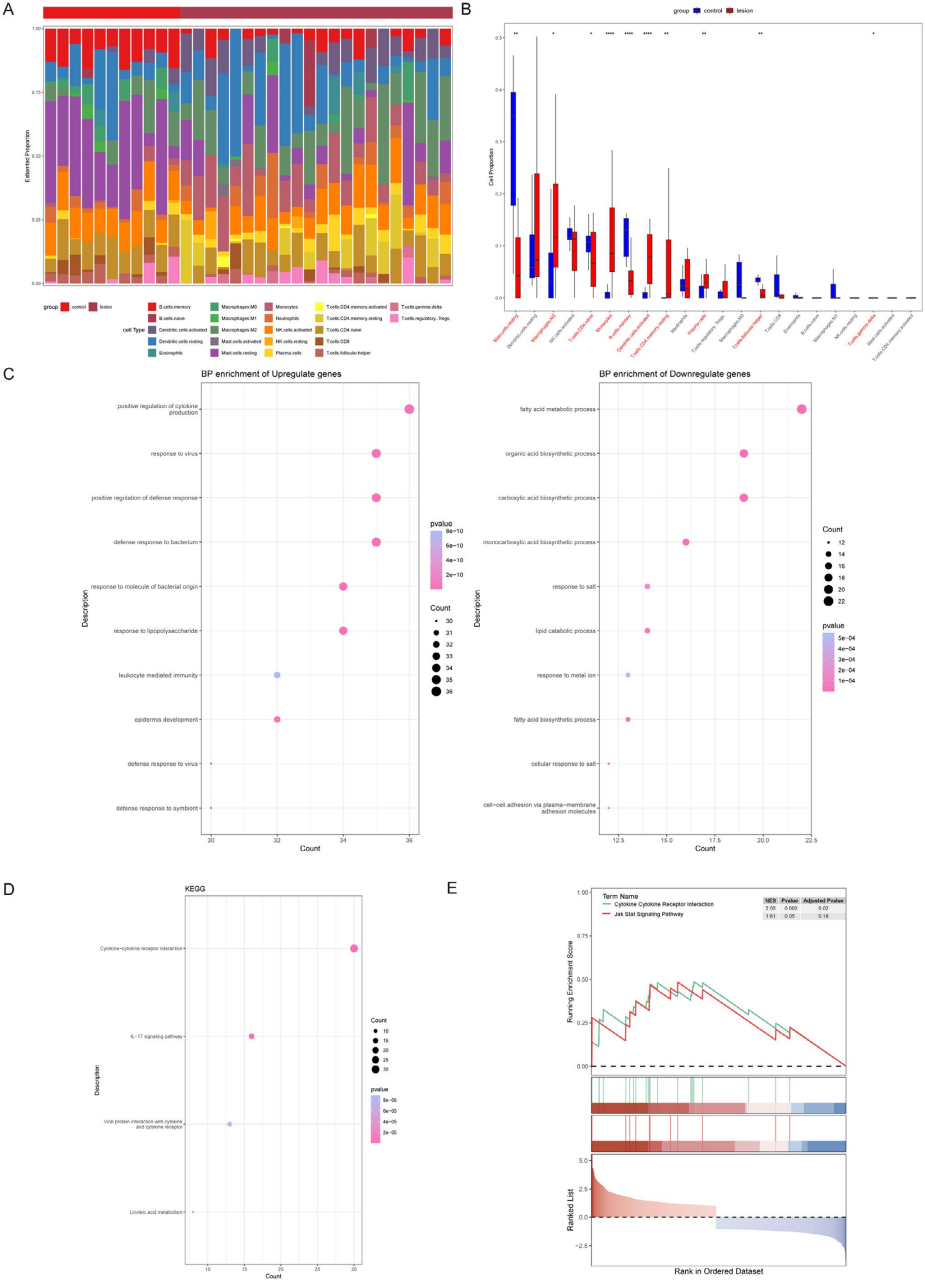
Immune infiltration and enrichment analysis of DEGs in ad. (A) The stacked histogram shows the distribution of 22 immune cell infiltrations between ad lesions and controls. (B) The boxplot of the proportions of 22 immune cell types reveals the differences in immune cell proportions between ad lesions and controls. (C) GO (D) KEGG, and (E) GSEA analyses of DEGs between AD lesions and controls. *p < 0.05, **p < 0.01, ****p < 0.0001.

As atopic dermatitis is a classic inflammatory skin disease, we conducted Immune infiltration analysis on lesional skin DEGs. First, we quantified the levels of immune cell infiltration and presented the proportions and differences of 22 types of immune cell infiltrates in histogram form (Figure 1A). The histogram of immune cell infiltration differences showed that the abundance of monocytes, M2 macrophages, resting CD4+ memory T cells, activated dendritic cells (DCs) and plasma cells was significantly higher in the lesional skin compared to the control group, while resting mast cells, activated NK cells, naive CD4+ T cells, memory B cells, and M0/M1 macrophages showed lower infiltration (Figure 1B).

We then used the ‘clusterProfiler’ package in R to perform GO and KEGG enrichment analyses to explore the potential biological functions of the DEGs. The results indicated that the biological processes of upregulated genes were concentrated in the positive regulation of cytokine production and defence responses against bacteria, viruses and lipopolysaccharides, while downregulated genes were associated with fatty acid metabolism, carboxylic acid biosynthesis and organic acid biosynthesis (Figure 1C). KEGG analysis showed that the interaction between cytokine‐cytokine receptor interaction was the most significantly enriched pathway (Figure 1D), and the prominent differential signalling pathway identified by GSEA analysis was also cytokine –cytokine receptor interaction (Figure 1E).

**FIGURE 2:**
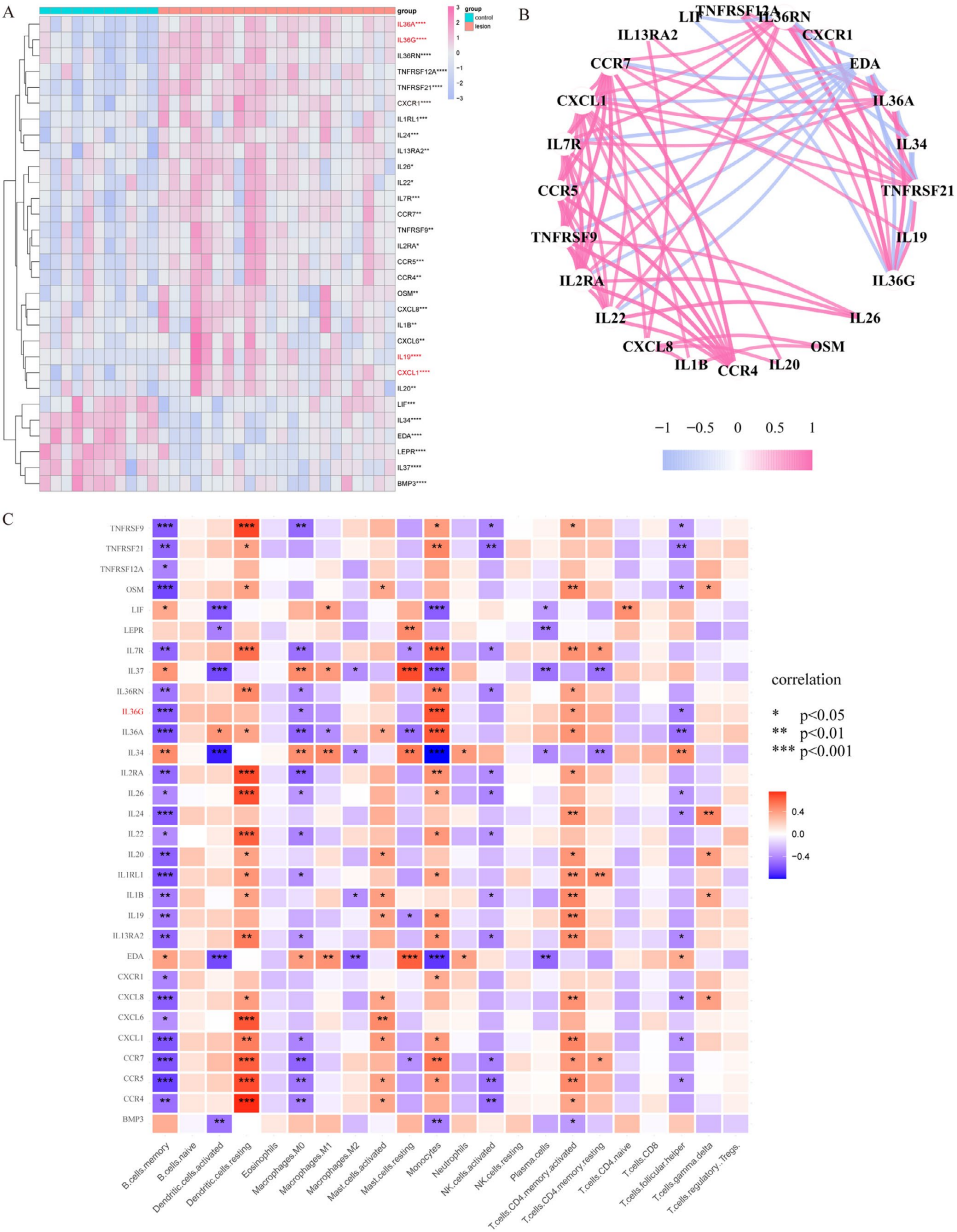
IL‐36G‐expressing monocytes may be a hub target in AD. (A) 30 differentially expressed cytokines and cytokine receptors between AD lesions and controls. (B) Co‐expression network of 30 cytokines and cytokine receptors. (C) The correlation between 30 cytokines and cytokine receptors and 22 immune cells. *p < 0.05, **p < 0.01, ***p < 0.001, ****p < 0.0001.

We extracted 30 differentially expressed cytokines and their receptors from the most significantly enriched pathways. Among these genes, IL36A, IL36G, IL19 and CXCL1 showed significant differences with *p* < 0.0001 (Figure 2A). Analysing the co‐expression relationships among these 30 genes revealed a good correlation between them (Figure 2B), suggesting their strong involvement in the pathogenesis of atopic dermatitis. Finally, we further investigated the correlation of cytokines and their receptors with immune cells, finding that IL‐36G was highly expressed specifically in monocytes (*p* < 0.001, Figure 2C), indicating that IL‐36G‐expressing monocytes may be a core target in atopic dermatitis.

### 3.2 IL‐36G‐Expressing Monocytes Are Also Highly Expressed in Psoriasis

**FIGURE 3:**
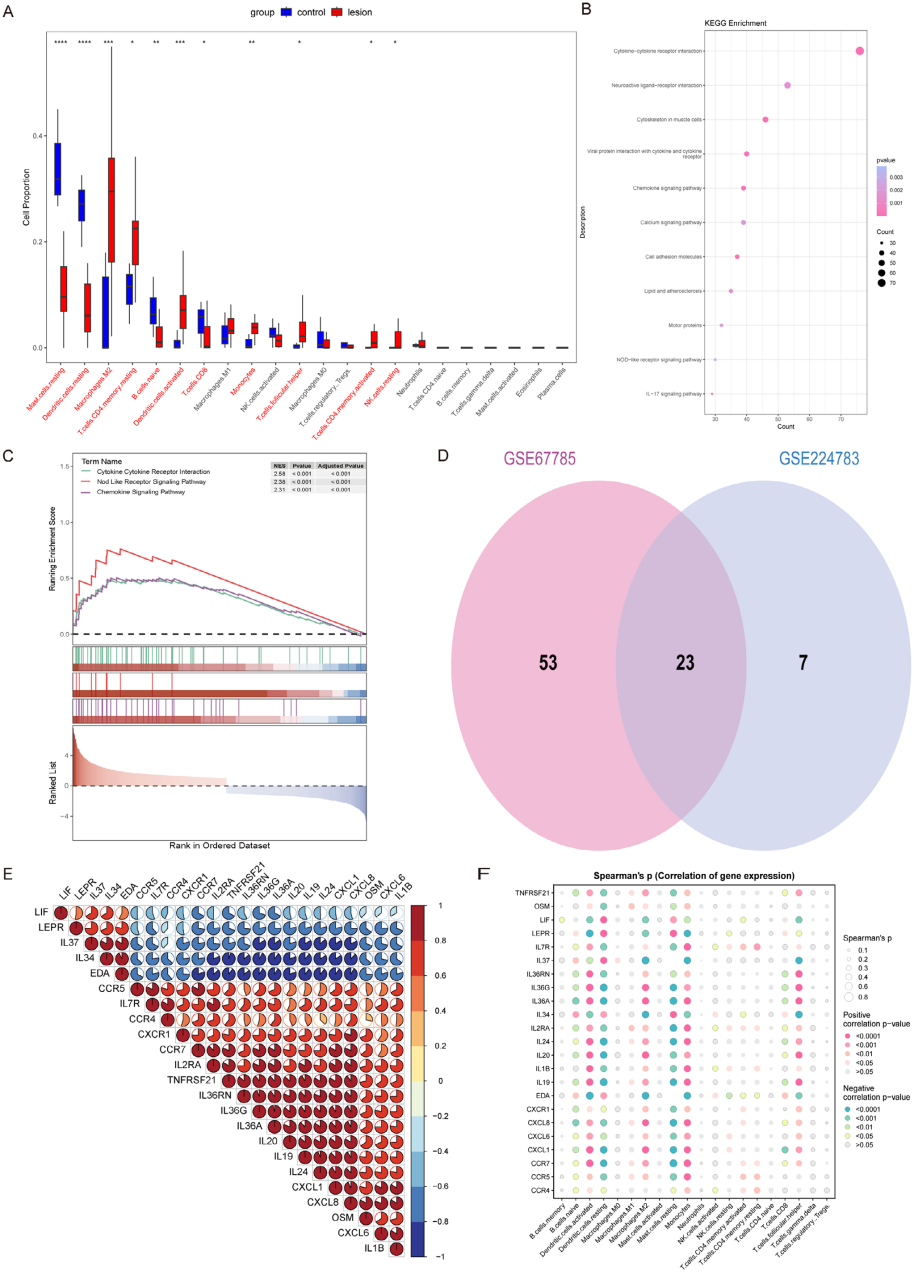
IL‐36G‐expressing monocytes are also highly expressed in PSO. (A) The boxplot of the proportions of 22 immune cell types reveals the differences in immune cell proportions between PSO lesions and controls. (B) KEGG and (C) GSEA analyses of DEGs between PSO lesions and controls. (D) Venn diagram showing 23 overlapping genes associated with AD and PSO's cytokines and cytokine receptors. (E) Co‐expression of 23 common cytokines and cytokine receptors. (F) The correlation between 23 common cytokines and cytokine receptors and 22 immune cells. *p < 0.05, **p < 0.01, ***p < 0.001, ****p < 0.0001.

From the GSE67785 dataset, we identified 1848 DEGs in psoriatic skin, including 882 upregulated genes and 966 downregulated genes. Next, we similarly studied the immune infiltration in psoriasis and found that M2 macrophages, monocytes, and resting CD4+ memory T cells were significantly elevated in psoriasis, similar to the findings in atopic dermatitis, while resting mast cells were reduced (Figure 3A).

GO analysis of the differential genes in psoriasis showed that the functions of upregulated genes were enriched in the positive regulation of cytokines, while downregulated genes were focused on the muscular system and membrane potential (Figure S1A). KEGG and GSEA analyses revealed that the cytokine and cytokine receptor pathways were also the most significantly enriched signalling pathways (Figure 3B,C). We extracted the genes from these pathways and found an intersection with atopic dermatitis, identifying 23 common cytokines and receptors (Figure 3D). IL36G was also highly expressed in psoriasis, and correlation analysis indicated that IL36G was closely related to IL36A, IL19, CXCL1, and CXCL8, all of which participate in the pathogenesis of psoriasis (Figure 3E).

Finally, we examined the correlation and expression of these cytokines and their related receptors in immune cells, discovering that monocytes had the highest positive correlation with these genes, with IL‐36G‐expressing monocytes also showing high expression in psoriasis (Figure 3F). Through immune cell‐related analysis, we found that IL36G‐expressing monocytes may be a common target in both atopic dermatitis and psoriasis.

### 3.3 IL‐36G Is Expressed but Is Not Associated With Monocytes in Chronic Urticaria

**FIGURE 4:**
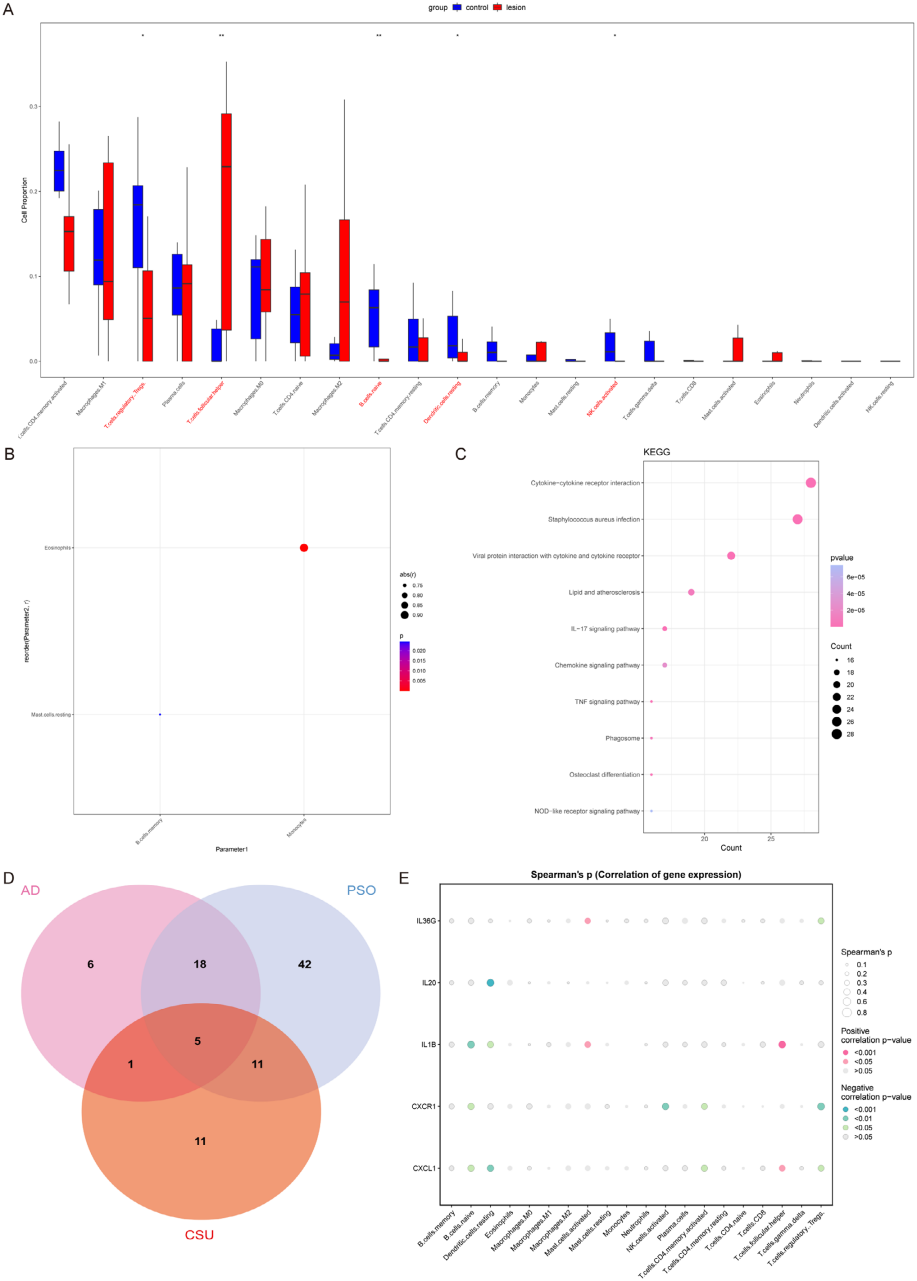
IL‐36G is expressed but is not associated with monocytes in CU. (A) The boxplot of the proportions of 22 immune cell types reveals the differences in immune cell proportions between CU lesions and controls. (B) The correlation of immune cells. (C) KEGG analyses of DEGs between CU lesions and controls. (D) Venn diagram showing 5 overlapping genes associated with AD, PSO, and CU's cytokines and cytokine receptors. (E) The correlation between 5 cytokines and cytokine receptors and 22 immune cells. *p < 0.05, **p < 0.01.

Next, to investigate the similarities and differences between chronic urticaria, psoriasis, and atopic dermatitis, we screened the DEGs in lesional and non‐lesional skin tissues of patients with CU, identifying 597 DEGs, including 420 upregulated genes and 177 downregulated genes. The immune infiltration results showed significant infiltration of helper T cells in urticaria, while Treg cells, naive B cells, resting dendritic cells (DCs), and activated NK cells were reduced in abundance (Figure 4A). Correlation analysis of immune cells revealed a positive correlation between monocytes and eosinophils in urticaria (Figure 4B). KEGG analysis showed significant enrichment in the cytokine and cytokine receptor signalling pathways, encompassing 28 genes (Figure 4C). We identified five cytokines and receptors that intersected between urticaria, atopic dermatitis, and psoriasis: IL36G, IL20, IL1B, CXCR1, and CXCL1 (Figure 4D). Correlation analysis with immune cells revealed that IL‐36G was positively correlated with activated mast cells in CU but showed no correlation with monocytes (Figure 4E). This result suggests that IL‐36G‐expressing monocytes may be important targets for distinguishing between atopic dermatitis/psoriasis and chronic urticaria.

### 3.4 IL‐36G‐Expressing Monocytes Also Show Differential Expression in Atopic Dermatitis and Psoriasis

**FIGURE 5:**
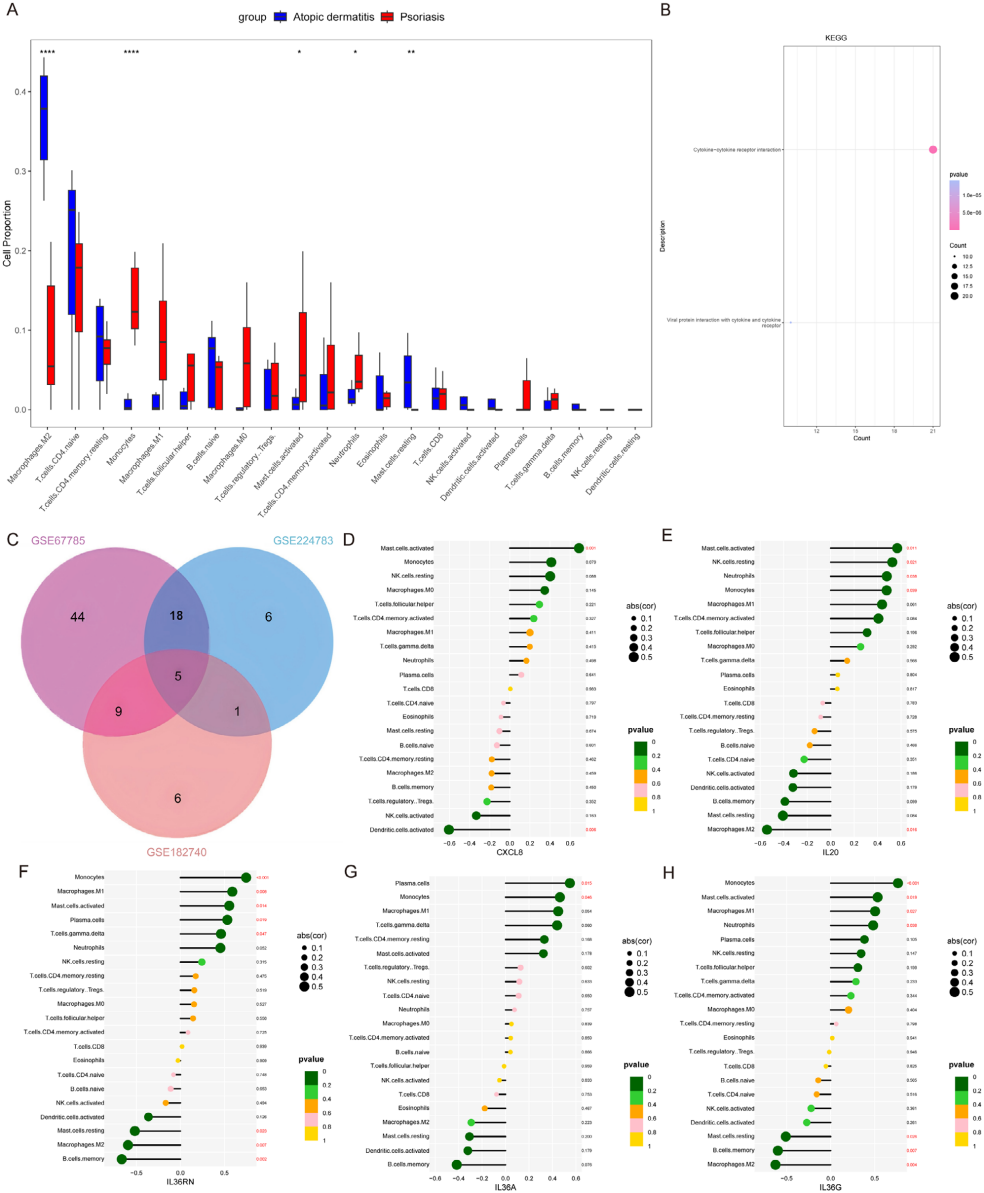
IL‐36G‐expressing monocytes also show differential expression in AD and PSO. (A) The boxplot of the proportions of 22 immune cell types reveals the differences in immune cell proportions between AD and PSO lesions. (B) KEGG analyses of DEGs between AD and PSO lesions. (C) Venn diagram showing 5 overlapping genes. (D–H) The lollipop diagram shows the correlation between CXCL8, IL20, IL36RN, IL36A, IL36G genes and 22 kinds of immune cells, and red marks indicate *p* < 0.05. *p < 0.05, **p < 0.01, ****p < 0.0001.

We further investigated the DEGs and cytokines in atopic dermatitis and psoriasis, identifying 466 DEGs from GSE182740, which included 133 upregulated genes and 333 downregulated genes. Immune infiltration analysis showed that the most significant differences in abundance were again observed in M2 macrophages and monocytes in both conditions. M2 macrophages were elevated in atopic dermatitis, while monocyte infiltration increased in psoriasis (Figure 5A).

After screening multiple groups, we identified five common differential cytokines (Figure 5B,C) and examined the relationship between immune infiltration and these five genes. The results indicated that IL‐36G had the highest correlation with monocytes (*p* < 0.001), and the other three cytokines were also correlated with monocytes (Figure 5D–H). These findings suggest that the central gene may influence the immunological features of atopic dermatitis and psoriasis, particularly regarding monocyte abundance.

### 3.5 Drug Prediction

**FIGURE 6:**
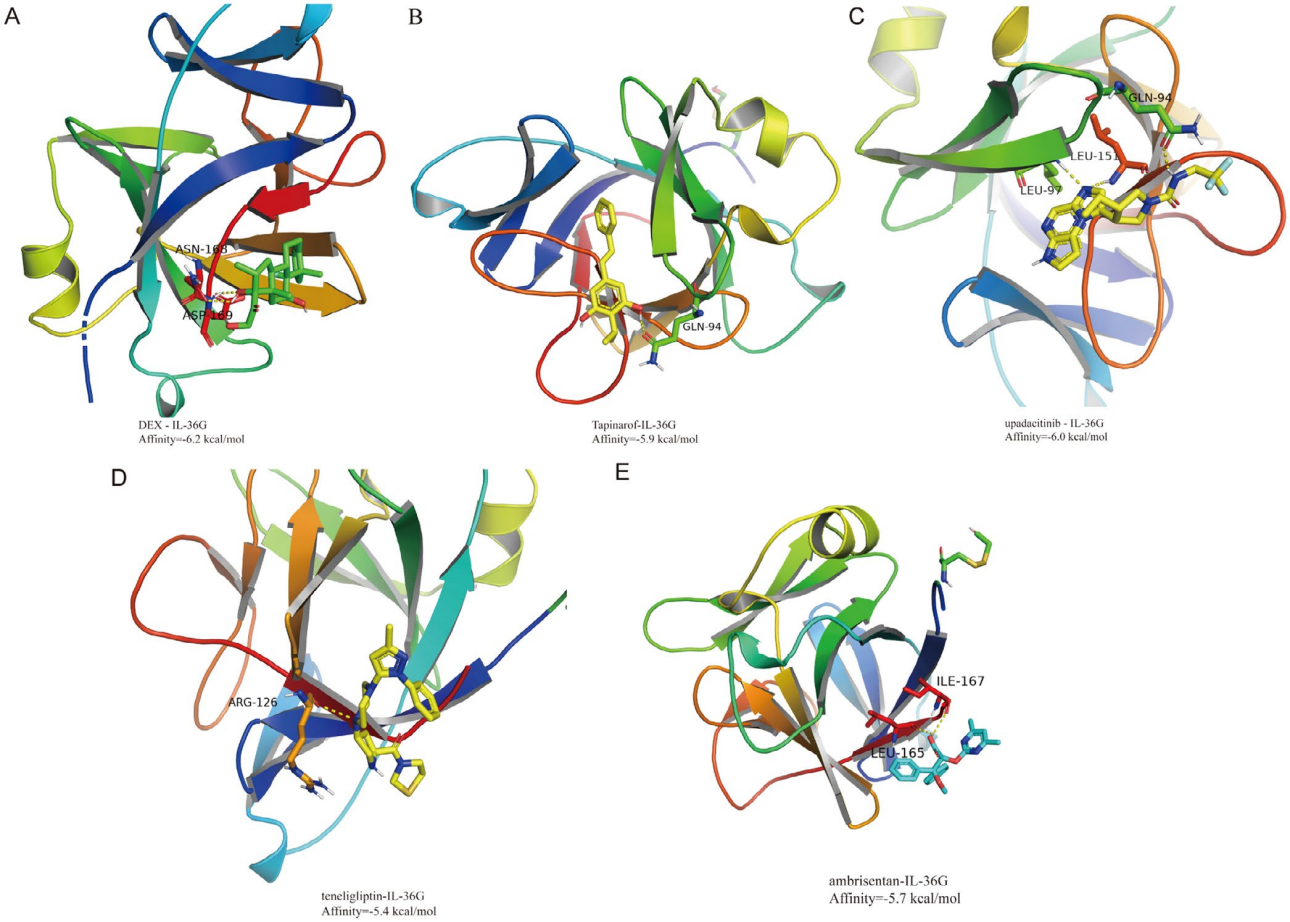
Molecular docking of therapeutic drugs. (A) Binding conformation of IL‐36G and Dexamethasone (binding energy = −6.2 kcal/mol). (B) Binding conformation of IL‐36G and Tapinarof (binding energy = −5.9 kcal/mol). (C) Binding conformation of IL‐36G and Upadacitinib (binding energy = −6.0 kcal/mol). (D) Binding conformation of IL‐36G and Ambrisentan (binding energy = −5.7 kcal/mol). (E) Binding conformation of IL‐36G and Teneligliptin (binding energy = −5.4 kcal/mol).

We performed molecular docking of clinical drugs and IL‐36G using three different therapeutic drugs for atopic dermatitis and psoriasis to verify IL‐36G as a key target for treating these conditions. The docking of glucocorticoid dexamethasone, aryl hydrocarbon receptor agonist Tapinarof, and Janus kinase (JAK) inhibitor Upadacitinib with IL‐36G showed that their affinities were all less than −5.0 kcal/mol. This indicates high affinity and suggests that these drugs may exert therapeutic effects on atopic dermatitis by targeting IL‐36G (Figure 6A–C). Finally, the atopic dermatitis‐specific DEGs were input into the cMAP database to screen the two small‐molecule drugs most highly correlated with atopic dermatitis, identified as Ambrisentan and Teneligliptin, which are considered an Endothelin receptor antagonist and a Dipeptidyl peptidase inhibitor, respectively. Docking of Ambrisentan and Teneligliptin with IL‐36G confirmed their binding energies were also less than −5.0 kcal/mol (Figure 6D,E), demonstrating that these small‐molecule drugs can bind to IL‐36G and exert potential therapeutic effects, making them promising candidates for treating atopic dermatitis and psoriasis.

## 4 Discussion

Through mining public transcriptome databases and applying a series of integrative biological methods, we identified a specific group of immune cells and key targets in atopic dermatitis, providing new insights for clinical diagnosis and treatment.

The IL‐36 axis is a critical component of the skin inflammation process, playing a pivotal role in various inflammatory skin diseases [6], involved in both innate and adaptive immune responses. In the skin, the cellular sources of IL‐36 cytokines include keratinocytes as well as immune cells. IL‐36 gene expression has been detected in human monocytes and macrophages [24]. Notably, IL‐36G expression has been identified within monocytic infiltrates of psoriatic skin lesions [25], further implying the important role of IL‐36G‐expressing monocytes in mucosal inflammation. However, there is currently a lack of research on the expression of monocytes and their cytokines in inflammatory skin diseases, particularly in lesional skin of atopic dermatitis. In our study using the GSE224783 dataset, we identified 997 DEGs in atopic dermatitis. Immune infiltration analysis revealed that immune cells, particularly M2 macrophages and monocytes, were concentrated in the lesional skin. GO analysis showed that the biological processes were centred around cytokine production. KEGG and GSEA enrichment analyses indicated that cytokine and cytokine receptor pathways are closely associated with atopic dermatitis. By extracting 30 core genes from these pathways, we identified IL‐36‐related cytokines. Correlation analysis between cytokines and immune cells confirmed that IL‐36G is specifically expressed in monocytes in atopic dermatitis, thereby identifying specific targets for atopic dermatitis.

We analysed the role of IL‐36G and monocytes in inflammatory skin diseases clinically similar to ad. ad and PSO are common chronic inflammatory diseases that exhibit directional associations with each other [26], and share genetic profiles, immune pathways, pathological changes, and comorbidities [27]. The shared characteristics of ad and PSO include increased epidermal hyperplasia and the infiltration of T cells and dermal dendritic cells (DCs) into the skin [28], as well as epidermal barrier disruption and impaired terminal differentiation of keratinocytes in lesional skin [29, 30]. In psoriasis, IL‐36G is primarily found in keratinocytes and is also expressed in infiltrating monocytes [24]. Using the same bioinformatics approach, we confirmed that M2 macrophages and monocytes are similarly elevated in psoriasis. Cytokine‐related pathways were found to be significantly enriched in psoriasis. Further analysis revealed that IL‐36G‐expressing monocytes are highly expressed in psoriasis, indicating their potential as a shared therapeutic target for both atopic dermatitis and psoriasis. Moreover, differential gene expression analysis between atopic dermatitis and psoriasis demonstrated higher levels of IL‐36G and monocytes in psoriasis.

ad and CU are both type 2 inflammatory skin diseases, accompanied by severe itching that affects both children and adults [31]. Itch stimuli and disruptions to skin barrier integrity trigger epithelial stress responses, leading keratinocytes to release epithelial‐derived cytokines [32]. Cytokines, including IL‐4, IL‐13, TSLP, IL‐31, IL‐25, and IL‐33 [33, 34], are released by mast cells, group 2 innate lymphoid cells, keratinocytes, and type 2 T lymphocytes. These cytokines serve as key regulators of chronic itch [35]. Type 2 immune responses and cytokines play a prominent role in the pathophysiology of chronic pruritic skin diseases [35], mediating the clinical manifestations of type 2 inflammatory skin disorders. Previous studies have confirmed the critical role of IL‐36G and IL‐36G‐expressing monocytes in ad. However, there is currently a lack of research on their role in CU. Our findings revealed that although IL‐36G is highly expressed in both diseases, monocytes do not show significant differences in CU. This suggests that IL‐36G‐expressing monocytes may serve as a key target for distinguishing between ad and CU.

Although the mechanisms of ad and PSO differ, most conventional systemic therapies, including immunosuppressants and phototherapy, are effective for both conditions. Target‐specific drugs, such as JAK inhibitors and PDE4 inhibitors, can also be used safely and effectively for both diseases. Tapinarof is a small‐molecule aryl hydrocarbon receptor (AhR) agonist clinically used to treat psoriasis and atopic dermatitis [36]. Upadacitinib is a selective Janus kinase (JAK) inhibitor that effectively inhibits JAK1, thereby suppressing cytokine signalling pathways critical to inflammatory diseases. It is used to treat chronic inflammatory skin diseases, including ad and PSO [37]. We conducted molecular docking analysis of DEX, Tapinarof, and Upadacitinib with IL36G and found that these drugs exhibit strong binding affinity. This suggests that they may exert their effects by inhibiting IL36G, thereby supporting the feasibility of targeting IL36G in inflammatory skin diseases. Additionally, CMap analysis predicted potential small‐molecule drugs capable of reversing the expression of DEGs in ad. Validation studies revealed that the predicted drugs also demonstrated strong binding to IL36G. Topical application of ambrisentan has been shown to alleviate histological inflammation and cytokine expression in lesional skin of mice [38], while teneligliptin reduces the expression of pro‐inflammatory cytokines [39].

This study, based on bioinformatics and immune infiltration analysis, identified IL36G‐expressing monocytes as a critical target in atopic dermatitis (ad) and an important player in psoriasis. IL36G‐expressing monocytes are specifically expressed in both psoriasis and ad, and they can serve as a distinguishing target between urticaria and ad, two type 2 inflammatory skin diseases. However, this study has some limitations. Firstly, the number of transcriptome datasets for atopic dermatitis in our study is limited, and no additional datasets were used to validate the analysis results. Finally, the biological function and molecular mechanisms of IL36G monocytes in atopic dermatitis require further investigation, and the efficacy of the two predicted IL36G‐targeting drugs also awaits validation.

This study identified IL36G‐expressing monocytes as a potential target for ad and demonstrated through immune infiltration analysis that ad shares similarities with psoriasis rather than urticaria. IL36G‐expressing monocytes serve as a common biomarker for ad and psoriasis and can also be used as a distinguishing target between ad and urticaria. These findings provide a novel therapeutic target for the clinical treatment of ad.

## Author Contributions

**Yitao Yang:** conceptualization (equal), data curation (lead), formal analysis (lead), investigation (equal), methodology (equal), software (lead), validation (equal), writing – original draft (lead), writing – review and editing (lead). **Lei Wang:** conceptualization (equal), investigation (equal), methodology (equal), writing – review and editing (equal). **Longmei Yu:** writing – original draft (supporting). **Chenxi Chang:** writing – original draft (supporting). **Honglei Zhang:** writing – original draft (supporting). **Linhan Hu:** writing – original draft (supporting). **Juntong Liu:** writing – original draft (supporting). **Yihang Zhang:** software (supporting), writing – original draft (supporting). **Hui Han:** writing – original draft (supporting). **Haiyun Zhang:** funding acquisition (supporting), resources (equal), supervision (equal). **Yumei Zhou:** funding acquisition (supporting), resources (equal), supervision (equal). **Ji Wang:** funding acquisition (lead), project administration (equal), resources (equal).

## Ethics Statement

This study was conducted in accordance with local legislation and institution requirements.

## Conflicts of Interest

The authors declare no conflicts of interest.

## Supporting information


**Figure S1.** Enrichment analysis of DEGs in PSO. GO analyses of (A) upregulate and (B) downregulate DEGs between PSO lesions and controls.

## Data Availability

The datasets (GSE224783, GSE67785, GSE72540 and GSE182740) that support the findings of this study are openly available in the GEO database (GSE224783: https://www.ncbi.nlm.nih.gov/geo/query/acc.cgi?acc=GSE224783, GSE75819: https://www.ncbi.nlm.nih.gov/geo/query/acc.cgi?acc=GSE67785, GSE72540: https://www.ncbi.nlm.nih.gov/geo/query/acc.cgi?acc=GSE72540, GSE182740: https://www.ncbi.nlm.nih.gov/geo/query/acc.cgi?acc=GSE182740).
