# Supplementary figures and images for: Monocytes Expressing IL‐36G Play a Crucial Role in Atopic Dermatitis
Source: J Cell Mol Med. 2025 Mar 30;29(7):e70503. doi: 10.1111/jcmm.70503 (PMC11955416; doi:10.1111/jcmm.70503)

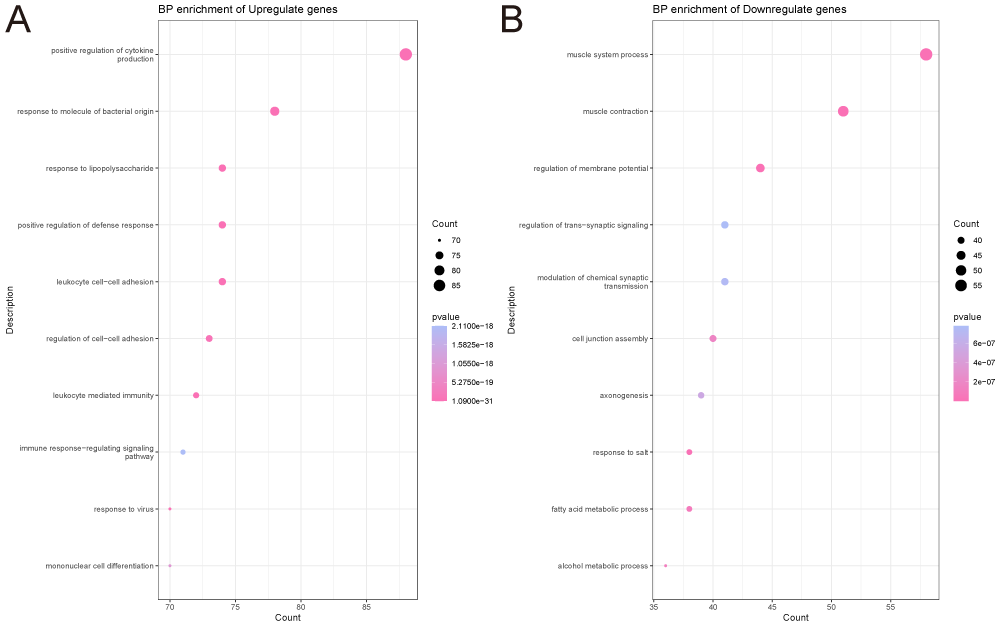

Supplement: Supplementary file 1 — Figure S1. Enrichment analysis of DEGs in PSO. GO analyses of (A) upregulate and (B) downregulate DEGs between PSO lesions and controls. [file JCMM-29-e70503-s001.tif]
